# Ultrasound-Based Anatomical Assessment of the Most Common Shoulder Soft Tissue Injuries in Young Adults

**DOI:** 10.3390/healthcare13161984

**Published:** 2025-08-13

**Authors:** Carlos Miquel García-de-Pereda-Notario, Luis Palomeque-Del-Cerro, Ricardo García-Mata, María Rodriguez-Isarn, Hugo Rodriguez-Isarn, Luis Alfonso Arráez-Aybar

**Affiliations:** 1 Department of Anatomy and Embryology, Faculty of Medicine, Universidad Complutense de Madrid, 28040 Madrid, Spain; arraezla@med.ucm.es; 2 UCM Research Group No. 920202, Faculty of Medicine, Universidad Complutense de Madrid, 28040 Madrid, Spain; 3 Department of Physiotherapy, Faculty of Nursing and Physiotherapy “Salus Infirmorum”, Universidad Pontificia de Salamanca, 28015 Madrid, Spain; lpalomequede@upsa.es; 4 Escuela de Osteopatía de Madrid, 28033 Madrid, Spain; 5 Research Support, UCM IT Services, 28040 Madrid, Spain; 6 Department of Rehabilitation and Movement, Universidad Nacional de San Martín, Buenos Aires B1650, Argentina; m.isarn@escuelaosteopatiamadrid.com (M.R.-I.);; 7 Escuela de Osteopatía de Madrid, Buenos Aires B1602, Argentina

**Keywords:** shoulder injuries, ultrasound diagnosis, shoulder ultrasound, soft tissue shoulder injuries, shoulder pain etiology

## Abstract

**Introduction:** Shoulder pain is one of the leading causes of medical consultation, highlighting the need to identify the most frequently affected tissues to improve diagnosis. This study aims to determine the most common shoulder soft tissue injuries in young adults using musculoskeletal ultrasound (US). **Methods:** An observational cross-sectional study was conducted with 66 individuals aged 18 to 45 years; 35 participants reported shoulder pain and 31 did not. All participants received shoulder US by a specialist. Structures analyzed included the rotator cuff tendons, the long head of the biceps tendon (LHBT), and the subacromial–subdeltoid bursa. **Results:** The supraspinatus tendon was the most frequently affected structure, accounting for 65.1% of clinical findings, and its involvement was strongly associated with subscapularis tendinitis (OR = 18.45). The subscapularis tendon represented 31.8%, tenosynovitis of the LHBT occurred in 7.6%, and the subacromial–subdeltoid bursa was affected in 1.5%. Cluster analysis revealed three distinct profiles based on age, pain status, and tendon involvement: Cluster 1 (*n* = 23; mean age 21.6 ± 3.8 years) included younger individuals with minimal pain and tendinopathy (21.7%); Cluster 2 (*n* = 21; mean age 33.6 ± 2.6) consisted of intermediate-age participants with moderate pain and predominant supraspinatus tendinitis (71.4%); and Cluster 3 (*n* = 22 mean age 42.1 ± 1.6) comprised older individuals with the highest prevalence of pain and combined tendon lesions (81.8%). **Conclusions:** This study confirms the clinical value of musculoskeletal US in detecting soft tissue injuries, including subclinical findings. The supraspinatus tendon was the most frequently affected structure, often associated with subscapularis tendinitis and other combined lesions in older individuals. US proved useful in identifying distinct injury profiles based on age and pain status, supporting its role in early diagnosis and tailored management strategies.

## 1. Introduction

The shoulder joint is one of the most complex and mobile joints in the human body, essential for daily activities. This mobility comes at the cost of relative instability, making it prone to injuries that can significantly impact quality of life [1,2]. Historically, evaluating shoulder injuries has posed challenges, with traditional clinical methods limited in detecting soft tissue abnormalities. The introduction of advanced imaging, particularly musculoskeletal US, has improved diagnostic accuracy for soft tissue injuries [3,4].

Unlike other imaging modalities, US offers dynamic, real-time assessment of both symptomatic and asymptomatic patients, and is cost-effective and non-invasive [5,6,7].

Subclinical injuries can become symptomatic over time, making early detection essential. Shoulder pain is the third most common cause for medical consultation, affecting up to 67% of individuals during their lifetime [8,9].

The most commonly affected tissues in this joint include the rotator cuff tendons, the LHBT, the subacromial–subdeltoid bursa, and the joint capsule [10,11].

Among these, the supraspinatus, infraspinatus, teres minor, and subscapularis muscles play a critical role in shoulder stability and movement [12,13].

The subacromial–subdeltoid bursa is a synovial structure that facilitates smooth gliding of the rotator cuff tendons, particularly the supraspinatus, under the acromion during arm movements. It plays a key role in shoulder biomechanics, and its dysfunction can lead to mechanical impairment [12,14].

The LHBT plays a critical role not only in tendinopathies but also in shoulder surgery. Recent reviews describe innovative uses of the LHBT in reconstructive arthroscopic techniques, extending beyond tenotomy to tendon preservation and autograft for rotator cuff and instability procedures, highlighting its biomechanical and functional implications during postoperative recovery [15,16].

The LHBT contributes to elbow flexion, forearm supination, and shoulder stabilization. Its tendon is prone to conditions such as tenosynovitis, tendinitis, and superior labral injuries [17,18].

Shoulder pain occurs at any age, but is more common after 50 due to degeneration, while in younger adults, it is mainly linked to trauma or overuse [9,19].

The aging global population, along with increased physical activity among older adults, is expected to lead to a rise in shoulder pain cases linked to degenerative pathologies. Therefore, it is crucial to identify the most common injuries and standardize diagnostic procedures to accurately identify the anatomical structures of the shoulder complex responsible for pain, ensuring effective therapeutic interventions [20,21].

This study aims to determine the most frequently affected shoulder tissues in a population of young adults aged 18 to 45 years, using musculoskeletal US as a diagnostic tool. The findings provide evidence of the distribution of common injuries and support the development of more effective diagnostic, preventive, and therapeutic strategies aligned with current clinical needs.

While the diagnostic performance of musculoskeletal ultrasound has been widely investigated in athletic populations, evidence remains limited in broader young adult groups, particularly university students and healthcare professionals. Given the functional demands and clinical relevance of shoulder conditions in these populations, further research is warranted to characterize common soft tissue injuries and enhance early diagnostic strategies [22,23].

## 2. Materials and Methods

### 2.1. Ethics

The study was approved by the Ethics Committee of Hospital Clínico San Carlos in Madrid (reference: 22/148-E).

### 2.2. Study Design

An observational and cross-sectional study was conducted to identify the most frequent injuries in shoulder tissues using US evaluation. The study took place between April and December 2022 at the Radiology Department of Viamed Santa Elena Hospital in Madrid, Spain.

A total of 66 participants were included. Although no a priori sample size calculation was performed, this number is comparable to similar US-based diagnostic studies on shoulder pathology in young adults, and it was sufficient to detect statistically significant differences between groups. Post hoc analysis confirmed that the sample provided adequate statistical power (≥0.80) for detecting medium effect sizes (Cohen’s d ≈ 0.5) in comparisons of continuous variables, with a significance level of α = 0.05.

### 2.3. Study Population

Potential participants were contacted through healthcare professionals at Viamed Santa Elena Hospital and faculty members from the Department of Anatomy and Embryology at the Faculty of Medicine, Universidad Complutense de Madrid.

The procedure was divided into three phases:

In the first, informative phase, candidates received a document detailing the study, including inclusion and exclusion criteria.

- Inclusion criteria required participants to be residents of Spain, and aged between 18 and 45 years. They could either have shoulder pain, present a structural shoulder alteration detectable by ultrasound (e.g., tendinopathy, bursitis, or partial tendon tear), or be completely healthy.
- Exclusion criteria included having tumors, infections, recent upper limb or cervical spine surgery, vascular diseases, shoulder malformations, or fever at the time of the study.

In the second phase (screening), applying these criteria resulted in a final eligible sample of 66 individuals. In the third phase (voluntary enrollment), participants signed an informed consent form detailing the objectives and procedures of the study, signifying their formal and voluntary inclusion.

**Figure 1:**
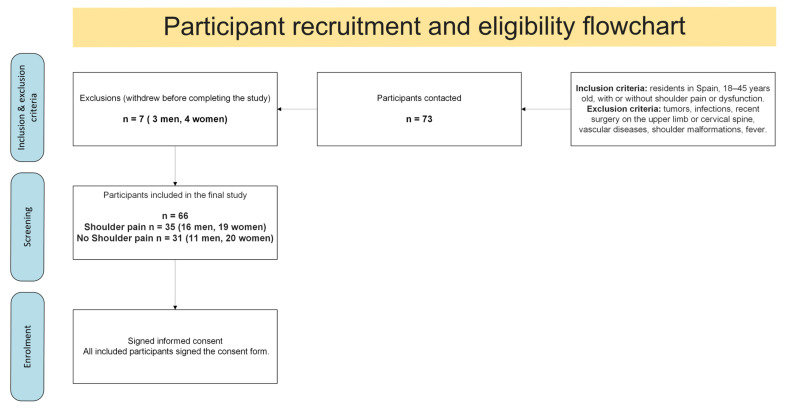
Flowchart of participant recruitment, eligibility criteria, exclusions, and final enrollment in the study.

Initially, 73 individuals aged 18 to 45 (30 men, 43 women) were recruited. Seven withdrew before completion (three men, four women), resulting in a final sample of 66 participants. A detailed flowchart illustrating the recruitment process, applied eligibility criteria, and final enrollment is provided in Figure 1.

All participants completed the McGill Pain Questionnaire prior to the US examination. This instrument includes a visual analog scale that allowed for the categorization of pain intensity across participants. These data were collected systematically for future analysis [24].

### 2.4. Procedure

After providing informed consent, all participants underwent a high-resolution musculoskeletal shoulder US (Canon CUS-AA000 Aplio a; Canon Medical Systems Corporation, Ōtawara, Japan), conducted by a certified radiologist with nine years of clinical experience.

The structures evaluated included the following: (A) supraspinatus tendon, (B) subscapularis tendon, (C) long head of the biceps tendon, (D) subacromial bursa, and (E) subdeltoid bursa. Findings such as tendinitis, tenosynovitis, calcifications, chronic degenerative tendinopathy, and fluid presence in bursae were recorded. Each participant received an individual clinical report issued by the hospital’s radiology service.

US examinations were performed according to the most widely accepted protocols in the scientific literature for measuring the acromiohumeral distance [13,14,15,16]. Participants were assessed in a seated position, with the trunk in a neutral flexion–extension posture and the arm resting alongside the body in an anatomical position. The sonographer placed the transducer on the anterolateral aspect of the shoulder and measured the shortest linear distance between the inferior border of the acromion and the superior aspect of the humeral head. The US system automatically calculated this distance in millimeters.

To assess measurement reliability, an intra-observer agreement study was conducted on a subsample of 15 participants. The same examiner performed three consecutive measurements per subject. All measurements were taken in a single session, within a time frame that did not exceed 15 min, to minimize intra-session variability and ensure consistency. The analysis yielded an intraclass correlation coefficient (ICC) of 0.95 (95% CI: 0.91 to 0.98), indicating excellent reproducibility. Although three measurements were taken for reliability analysis, only a single measurement per participant was used in the final analysis of the main study, given the high consistency of the US device and the demonstrated intra-observer agreement.

In addition to US, a standard anteroposterior shoulder radiograph was performed with the participant in a standing position, following the usual radiographic protocol. All imaging procedures adhered to the internal guidelines of Hospital Viamed Santa Elena and were carried out by the same professionals who conducted the subsequent measurements.

The full assessment session had an approximate duration of 15 min per participant. Data collection and imaging procedures were carried out over a period of up to 12 months for both study groups.

### 2.5. Statistical Analysis

Descriptive statistics were used to determine the frequency of shoulder soft tissue injuries. Categorical variables were presented as absolute and relative frequencies, and continuous variables as means ± standard deviation (SD).

In addition to descriptive and inferential statistics, a hierarchical cluster analysis was performed to identify natural groupings within the participant sample. The analysis included variables such as age, pain status, and the presence of supraspinatus and subscapularis tendinitis. Data preprocessing and clustering were conducted using Python 3.13.3 (scikit-learn and SciPy libraries). Ward’s method with Euclidean distance was used to construct the linkage matrix and generate a dendrogram. The number of clusters was determined by visual inspection of the dendrogram and by applying a distance-based cut-off to define three clinically meaningful groups.

## 3. Results

### 3.1. Demographic Characteristics

The overall gender distribution showed 27 men (40.9%) and 39 women (59.1%), indicating greater female participation. Regarding age groups, 29 participants (43.9%) were between 18 and 32 years old, while 37 participants (56.1%) were aged 33 to 45, showing greater participation from the older age group.

It is known that women are more likely to experience shoulder pain, especially post-menopause due to hormonal changes affecting soft tissue and cartilage composition [25].

A total of 66 individuals (*n* = 66) were evaluated, with a mean age of 29.6 years (±9.0). Of these, 35 participants (53.0%) reported shoulder pain (16 men, 19 women), while 31 participants (47.0%) did not report any shoulder issues (11 men, 20 women). The average age of participants was also compared between pain and no-pain groups. Those reporting shoulder pain were (32.5 ± 8.2 years) compared to asymptomatic individuals (26.7 ± 7.6 years)^1^.

A summary of the demographic characteristics of the study population is presented in Table 1. The table includes the total sample size, distribution by age range and sex, and the division of participants by presence or absence of shoulder pain. It also shows the mean age for symptomatic and asymptomatic individuals, highlighting differences across groups.

A detailed overview of shoulder dominance and the side evaluated during the ultrasound examination is provided in Table 2 and Table 3. Table 2 presents the frequency and percentage of participants with right or left shoulder dominance, while Table 3 shows the side (right or left) of the shoulder that was assessed in each case. This information enhances the interpretability and reproducibility of the findings by clarifying lateralization patterns within the sample.

### 3.2. Comparison of Symptomatic and Asymptomatic Participants

Clinical findings were observed in 85.7% of participants with pain and in 29.0% of those without pain, suggesting significant differences (*p* < 0.001). A comparative summary of clinical findings between symptomatic and asymptomatic participants is provided in Table 4. The table highlights the percentage of individuals in each group presenting detectable clinical alterations, along with the associated statistical significance, reinforcing the observed disparity between groups.

### 3.3. Injury Frequency

The distribution of shoulder soft tissue injuries identified in the study sample is summarized in Table 5. The supraspinatus tendon was the most frequently affected structure, followed by the subscapularis tendon, with varying degrees of calcification, tendinopathy, and bursitis also observed. The table also includes the number of asymptomatic cases per injury type.

### 3.4. Comparison of Supraspinatus and Subscapularis Tendinitis

Table 6 presents the contingency analysis between the presence of tendinitis in the supraspinatus and subscapularis tendons. The chi-square test revealed a statistically significant association between both conditions, χ^2^(1) = 9.84, *p* < 0.001. The strength of this association, assessed using Cramér’s V, was moderate (V = 0.386). In terms of clinical relevance, individuals with supraspinatus tendinitis were 18.45 times more likely to also present tendinitis in the subscapularis tendon (OR = 18.45), compared to those without supraspinatus involvement.

These findings suggest a non-random co-occurrence pattern between supraspinatus and subscapularis pathologies in the studied population, indicating a potential clinical linkage that may warrant further investigation.

### 3.5. Cluster Analysis

A hierarchical cluster analysis was conducted based on participants’ age, pain status, and presence of supraspinatus and subscapularis tendinitis. Three distinct clusters emerged, representing natural groupings within the sample.

Cluster 1 included mostly younger individuals with a low frequency of pain and tendon involvement. Cluster 2 was characterized by moderate age and higher rates of supraspinatus tendinitis. Cluster 3 contained older participants with the highest prevalence of pain and combined tendinopathies. The cluster analysis revealed three distinct groups:

- Cluster 1 included mostly younger individuals with a low frequency of pain and tendinopathies.
- Cluster 2 was composed of participants of intermediate age with moderate pain and a higher prevalence of supraspinatus tendinitis.
- Cluster 3 contained older individuals with the highest rates of pain and combined tendon pathologies.

The hierarchical cluster analysis identified three distinct participant groups based on the input variables. Cluster 1 included mostly younger individuals with a low frequency of pain and tendon involvement. Cluster 2 was characterized by participants of intermediate age and higher prevalence of supraspinatus tendinitis. Cluster 3 consisted of older individuals who exhibited the highest prevalence of pain and combined tendon pathologies (Table 7).

The cluster analysis contributes to understanding the clinical heterogeneity of shoulder injuries in young adults. The identification of distinct participant profiles suggests that age, pain, and tendinopathy patterns are interrelated. Cluster-based approaches may support the development of personalized diagnostic and therapeutic strategies in musculoskeletal clinical settings. Future research with larger samples is recommended to validate these groupings and explore their prognostic value.

**Figure 2:**
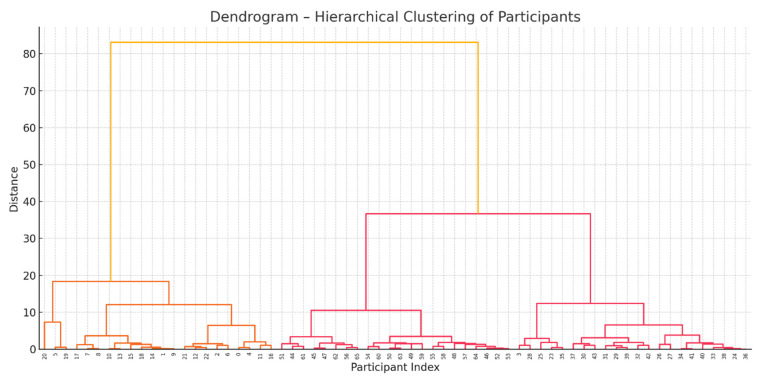
Dendrogram showing hierarchical clustering of participants. The color gradient (from red to yellow) shows similarity. Red indicates high similarity, and yellow low similarity. Distance is measured on the Y-axis.

The dendrogram in Figure 2 provides a visual representation of the hierarchical clustering process that led to the identification of three participant clusters. In this graph, the vertical axis (“Distance”) quantifies the dissimilarity between clusters, and the horizontal axis (“Participant Index”) identifies each individual. Visual analysis shows an initial main division (at an approximate distance of 85 units) that isolates a first group (orange branch). Subsequently, the remaining set bifurcates into two distinct clusters (pink branches) at a distance of around 47 units, with these three main divisions corresponding to the described clusters. The height of each joint in the dendrogram reflects the degree of dissimilarity at which participants or groups merged.

This dendrogram complements Table 5, which presents the specific characteristics of each cluster. Figure 2 graphically illustrates how these clusters were formed based on the input variables (age, pain, and supraspinatus and subscapularis tendinitis), thereby validating the appropriateness of the three-cluster solution as a faithful representation of the natural groupings within the sample.

### 3.6. Additional Findings

Approximately 18 participants (27.3%) had injuries in multiple structures. The most frequent combination was supraspinatus and subscapularis tendon involvement (24.2%, *n* = 16). Combinations involving the subscapularis and the LHBT, as well as all three tendons together, were observed in 3.0% (*n* = 2) of participants each.

## 4. Discussion

Notably, 29.0% (*n* = 9) of asymptomatic individuals presented US-detected injuries, demonstrating the tool’s capacity to identify early or subclinical lesions. High diagnostic concordance (85.7%) was found between US findings and clinical symptoms.

The results confirm that the supraspinatus tendon is the most commonly affected shoulder structure in young adults, followed by the subscapularis and LHBT. The subacromial–subdeltoid bursa was less clinically relevant.

These findings align with previous studies highlighting the supraspinatus’s anatomical vulnerability due to mechanical stress, especially in repetitive or strained movements [26].

These results have important clinical implications regarding the diagnostic value and applicability of musculoskeletal ultrasound in shoulder assessment.

Musculoskeletal US proved highly effective for diagnosing shoulder injuries, representing a particularly valuable tool in primary care and physiotherapy settings due to its real-time, non-invasive, and cost-effective assessment. This supports its use as a first-line diagnostic method, given its ability to rapidly identify soft tissue abnormalities such as tendinopathies, bursitis, or structural alterations.

US is a practical diagnostic tool in primary care centers and physiotherapy clinics, as it enables early diagnostic orientation and timely decision-making without the need for more expensive or less accessible imaging methods. This reinforces its role not only as a diagnostic modality but also as a practical solution for streamlining the evaluation and management of shoulder complaints in real-world clinical environments. Additionally, injuries were more prevalent in participants aged 33–45, suggesting a possible association with tissue aging and cumulative degeneration.

These findings also support the importance of early screening, even in mildly symptomatic or asymptomatic patients, given the detection of subclinical lesions in 29% of pain-free individuals. Moreover, the co-occurrence of supraspinatus and subscapularis tendinopathies highlights the value of comprehensive tendon evaluation.

Beyond clinical relevance, certain methodological considerations should be addressed.

Limitations include the cross-sectional nature of the study, which limits causal inference, and the exclusive use of US, which may miss deeper or complex pathologies. Future research could benefit from longitudinal designs and complementary imaging techniques such as X-rays [27].

It is important to note that the participants in this study were not representative of the general young adult population, as the sample was composed primarily of university students and healthcare professionals. Specifically, 42.8% (*n* = 18) of the participants were university students, while 57.1% (*n* = 24) were active healthcare professionals. This occupational composition may influence the prevalence and type of shoulder conditions identified, as these individuals may differ in terms of physical activity, ergonomic exposure, and health awareness compared to other young adults, such as those engaged in manual labor or athletic pursuits. Therefore, the findings should be interpreted within the context of this specific population.

The cluster analysis enhanced the understanding of clinical heterogeneity in young adults with shoulder conditions. The identification of three distinct participant profiles suggests that age and the presence of pain and tendinopathies follow specific combinatory patterns. Cluster-based exploration may prove useful in developing personalized diagnostic or therapeutic approaches in musculoskeletal clinical settings [28].

Interestingly, a notable proportion of asymptomatic participants also presented structural abnormalities detectable via US. This raises important considerations regarding the potential for overdiagnosis, especially in musculoskeletal imaging. While early identification of subclinical findings may be beneficial, it is equally essential to differentiate between clinically significant lesions and incidental, non-symptomatic changes. These results align with the previous literature reporting similar imaging findings in asymptomatic shoulders, emphasizing the need for a cautious interpretation of imaging data within the broader clinical context [29].

One limitation of this study was the absence of functional outcome measures, such as pain scales or shoulder-specific functional assessments. Although US provided valuable structural insights, the lack of complementary clinical data, such as patient-reported pain intensity or range of motion limitations, prevents a comprehensive understanding of how the observed anatomical findings translate into functional impairment. Including such measures would have allowed for a more integrated interpretation of the clinical relevance of each injury and a clearer correlation between imaging results and patient experience [20].

Another important limitation lies in the potential lack of correlation between US findings and those obtained through other commonly used imaging modalities, such as conventional radiography. In many cases, shoulder X-rays do not reveal abnormalities that sufficiently explain the patient’s symptoms, particularly when dealing with soft tissue injuries. Traditionally, radiography has been more widely used than US in routine clinical practice. However, studies like the one presented here suggest that this paradigm may need to be reconsidered, especially in primary care settings [30].

Incorporating musculoskeletal US into standard diagnostic protocols could offer earlier and more precise identification of clinically relevant findings, particularly in patients whose pain cannot be explained radiographically. This diagnostic gap may otherwise lead to underestimation of relevant pathologies or delayed therapeutic interventions. Consequently, while US provides valuable real-time insights into tendinous and bursal structures, its findings may not always align with radiographic interpretations, highlighting the need for integrative diagnostic approaches that consider both clinical and imaging data [30].

In postoperative settings, particularly in patients with persistent shoulder pain following rotator cuff repair, the use of musculoskeletal US presents specific challenges. Postsurgical anatomical alterations, such as scarring, residual fluid collections, or the integration of autografts like the LHBT, can complicate image interpretation and require advanced operator expertise. The complexity increases in procedures involving partial articular supraspinatus tendon avulsion repairs, where tendon augmentation with the LHBT has been described as a promising technique to improve tendon healing and joint function. In these scenarios, US must be carefully employed to differentiate between normal postoperative changes and pathological findings, which is crucial for guiding further treatment decisions. This highlights the importance of clinician experience and familiarity with both surgical techniques and sonographic patterns in the postoperative shoulder [31].

Moreover, musculoskeletal US demonstrates significant applicability in younger patients for whom standard imaging techniques, such as radiography or magnetic resonance imaging, may be limited due to patient-specific constraints or concerns about radiation exposure. In these scenarios, US serves as a valuable alternative, especially when previous surgical procedures have altered normal anatomy or when residual postoperative pain persists. Its ability to provide real-time, dynamic imaging without ionizing radiation makes it particularly advantageous in evaluating complex clinical presentations in younger populations [32].

## 5. Conclusions

Future research should aim to include larger and more diverse samples to validate the groupings identified in this study and explore their potential prognostic value. Addressing current limitations such as the cross-sectional design, the absence of functional outcome measures, and the lack of correlation with complementary imaging modalities like radiography would enhance the clinical applicability and interpretative depth of future findings [33].

This study identified the supraspinatus tendon as the most frequently injured tissue in individuals with shoulder pain, followed by the subacromial–subdeltoid bursa and the infraspinatus tendon. These findings underscore the need to routinely assess these structures during clinical evaluations.

This study reinforces the importance of identifying the most frequent soft tissue injuries to improve diagnostic and therapeutic strategies and supports US as a valuable clinical tool.

Musculoskeletal US proved to be a highly effective, non-invasive, and cost-efficient diagnostic tool for shoulder soft tissue injuries. Its ability to provide real-time, accurate diagnosis makes it essential for daily clinical practice, especially in resource-limited settings.

In addition to descriptive data, this study revealed a strong association between tendon injuries: individuals with supraspinatus tendinitis were 18.45 times more likely to also present subscapularis tendinitis. These findings help characterize injury patterns that may inform more focused diagnostic protocols.

A hierarchical cluster analysis identified three distinct participant profiles based on age, pain status, and tendon involvement. Cluster 1 (*n* = 23) included younger individuals with minimal symptoms and low prevalence of tendon lesions; Cluster 2 (*n* = 21) comprised participants of intermediate age with predominant supraspinatus involvement; and Cluster 3 (*n* = 22) consisted of older individuals with the highest frequency of pain and combined tendinopathies. This classification highlights the clinical heterogeneity of shoulder disorders and supports the design of tailored diagnostic and therapeutic strategies.

Despite limitations related to its cross-sectional design, the study provides relevant evidence to improve the diagnosis and management of shoulder pain. Future longitudinal studies are recommended to better explore causal relationships and risk factors.

In conclusion, this study reinforces the clinical utility of musculoskeletal US across diverse clinical settings such as primary care and contributes to a deeper understanding of common soft tissue injuries in the shoulder.

## Figures and Tables

**Table 1:** Demographic characteristics of the participants.

Variable	*n* (66)	%/Mean (±SD)
Male	27	40.9%
Female	39	59.1%
With shoulder pain	35	53.0%
Males with pain	16	45.7%
Females with pain	19	54.3%
Without shoulder pain	31	47.0%
Males without pain	11	35.5%
Females without pain	20	64.5%
Age 18–32 years	29	43.9%
Age 33–45 years	37	56.1%
Mean age (years)		29.6 (±9.0)
Mean age (with pain) ^1^		32.5 (±8.2)
Mean age (without pain) ^1^		26.7 (±7.6)
Ultrasound-detected lesions ≥ 1	39	59.1%
Detected with pain ^2^	30	85.7%
Detected without pain ^2^	9	29.0%
Subacromial Distance		7.4 (±0.7)
Distance with pain ^3^		7.3 (±0.6)
Distance without pain ^3^		7.6 (±0.7)

^1^ An independent samples *t*-test confirmed the difference to be statistically significant (t = 3.02, *p* = 0.004), suggesting a possible age-related predisposition to shoulder injury (Shoulder Pain vs. No Shoulder Pain). ^2^ An association between shoulder pain and US-detected lesions was assessed using the chi-square test, which revealed a statistically significant relationship (χ^2^ = 19.57, *p* < 0.001). Individuals with shoulder pain were significantly more likely to present with identifiable soft tissue injuries. ^3^ An independent samples t-test revealed no statistically significant difference in acromiohumeral distance between participants with and without shoulder pain; t(64) = 1.748, *p* = 0.085. The mean distance was slightly lower in the pain group (7.3 ± 0.6 mm) compared to the no-pain group (7.6 ± 0.7 mm), with a 95% confidence interval for the mean difference ranging from −0.040 to 0.602 mm. The subacromial distance, as measured by ultrasound as described in Section 2.4, was analyzed separately for participants with and without shoulder pain.

**Table 2:** Dominant shoulder side: frequencies and percentages.

Dominant Shoulder Side	Frequency	Percent
Right	*n* = 59	89.4%
Left	*n* = 7	10.6%

**Table 3:** Evaluated shoulder side: frequencies and percentages.

Evaluated Shoulder Side	Frequency	Percent
Right	*n* = 44	66.7%
Left	*n* = 22	33.3%

**Table 4:** Comparison of symptomatic and asymptomatic participants.

Group	Clinical Findings Observed (%)
With shoulder pain	85.7%
Without shoulder pain	29.0%

**Table 5:** Frequency of diagnosed shoulder tissue injuries.

Injury Type	Total Cases (*n*) (% Total)	Asymptomatic Cases (*n*) (% Total)
Supraspinatus tendinitis	35 (53.0%)	8 (12.1%)
Subscapularis tendinitis	15 (22.7%)	4 (6.1%)
Supraspinatus calcification	7 (10.6%)	1 (1.5%)
Subscapularis calcification	6 (9.1%)	1 (1.5%)
Long head of biceps tenosynovitis	5 (7.6%)	-
Supraspinatus tendinopathy	1 (1.5%)	-
Subacromial–subdeltoid bursitis	1 (1.5%)	-

**Table 6:** Association between supraspinatus and subscapularis tendinitis.

	Subscapularis NO	Subscapularis YES
Supraspinatus NO	29	1
Supraspinatus YES	22	14

**Table 7:** Summary of participant characteristics by cluster.

Cluster	Participants (*n*)	Mean Age (SD)	% with Pain	% with Supraspinatus Tendinitis	% with Subscapularis Tendinitis
1	23	21.6 ± 3.8	21.7	13.0	8.7
2	21	33.6 ± 2.6	71.4	71.4	28.6
3	22	42.1 ± 1.6	81.8	81.0	36.4

## Data Availability

The data that support the findings of this study are available from the corresponding author upon reasonable request.
